# Third-generation internet-based brief interventions for problem drinkers: how far can technology take us, and what types of drinkers can be reached?

**DOI:** 10.1186/1940-0640-7-S1-A90

**Published:** 2012-10-09

**Authors:** Trevor van Mierlo

**Affiliations:** 1 Rotman School of Management, University of Toronto, Toronto, Ontario, Canada

## 

For over a decade, a number of randomized controlled trials (RCTs) have found that internet-based brief intervention (IBBI) can reduce consumption in people with alcohol use disorders. As technology becomes increasingly sophisticated, IBBI has the potential to offer highly tailored feedback to different populations. First launched in 2005, CheckYourDrinking.net (CYD), version 3.0, is a free anonymous IBBI that has undergone several randomized controlled trials and technical upgrades. Apart from the free online version, the technology has been modified for several specific populations such as college students, youth aged 13-17, public health institutions, and for use as a screening tool in private clinics. To illustrate how algorithms can be modified, we are examining data from 21,640 Canadian men (59%) and women (41%) who anonymously accessed CheckYourDrinking.net version 2.0 from April 8, 2008, to July 28, 2010. Through describing CYD’s development methodology and research-based maturation process, we illustrate how IBBI can be tailored for special populations. Technical limitations and other barriers are discussed. The purpose of this presentation is to share knowledge of the resources required for the ongoing technical enhancement and maintenance of IBBIs. With the use of sophisticated algorithms, IBBIs hold the exciting potential of providing highly tailored feedback to not only people with problem drinking but for those with specific unhealthy drinking patterns (i.e., heavy episodic drinking) and for special populations across languages and cultures. In order to fully understand the potential of IBBI technology, further research is required.

